# Supplementary material for: Cross-link assisted spatial proteomics to map sub-organelle proteomes and membrane protein topologies
Source: Nat Commun. 2024 Apr 17;15:3290. doi: 10.1038/s41467-024-47569-x (PMC11024108; doi:10.1038/s41467-024-47569-x)
Supplement: Supplementary file 1 — Supplementary Information [file 41467_2024_47569_MOESM1_ESM.pdf]

Cross-link assisted spatial proteomics to map sub-organelle proteomes and membrane protein topologies

Ying Zhu *et al.*

– Supplementary Information –

Supplementary Figure 1

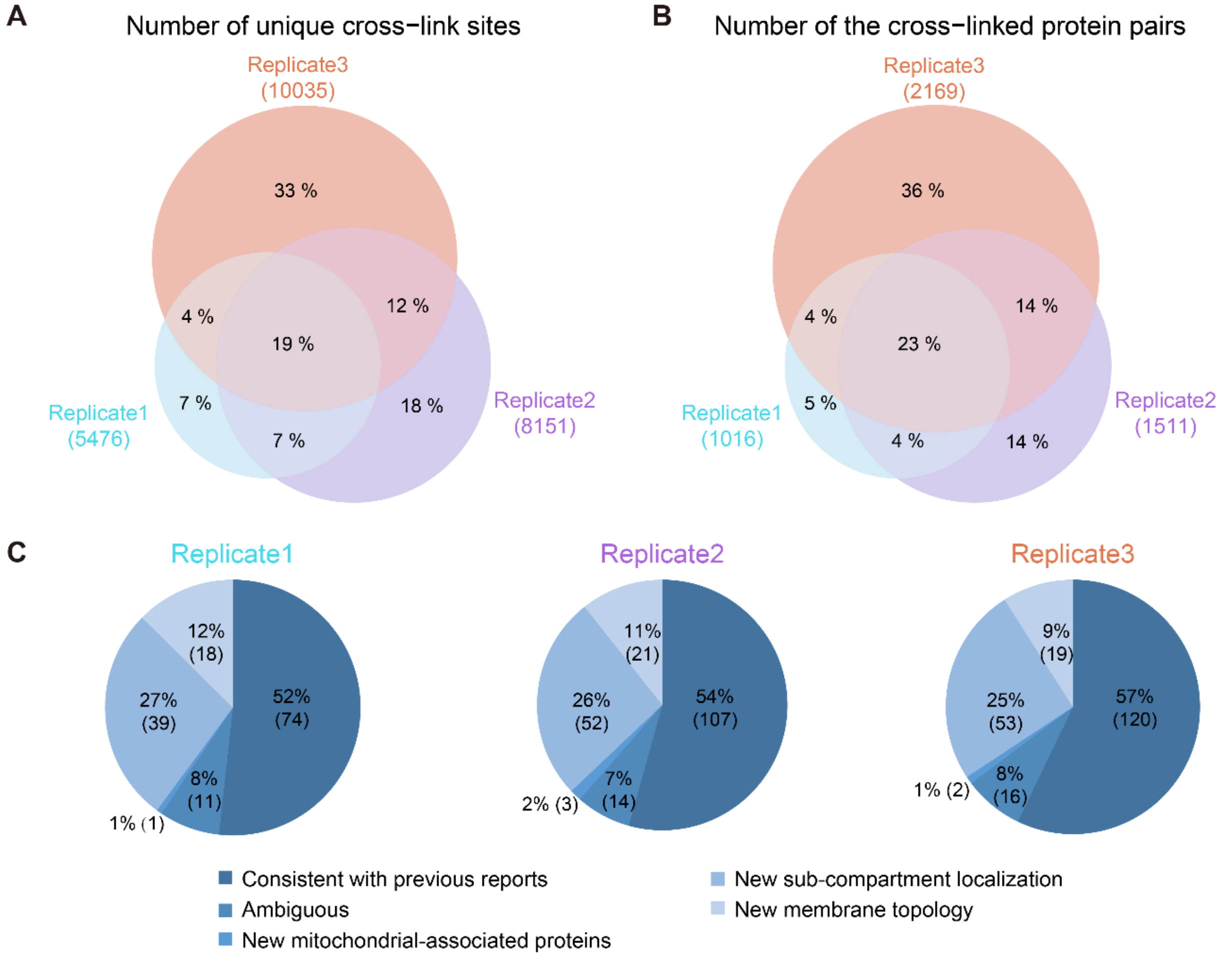

Supplementary Figure 1. CLASP performance in three biological replicates.

(**A**) Venn diagram of unique cross-link sites from three biological replicates. The results are in line with published XL-MS studies of eukaryotic systems, which typically report 10-20% overlap between three biological replicates at unique cross-link site level [1-4].

(**B**) Venn diagram of cross-linked protein pairs from three biological replicates.

(**C**) Distribution chart for five different categories after CLASP annotation in each biological replicate.

Source Data are provided as a Source Data file.

Supplementary Figure 2

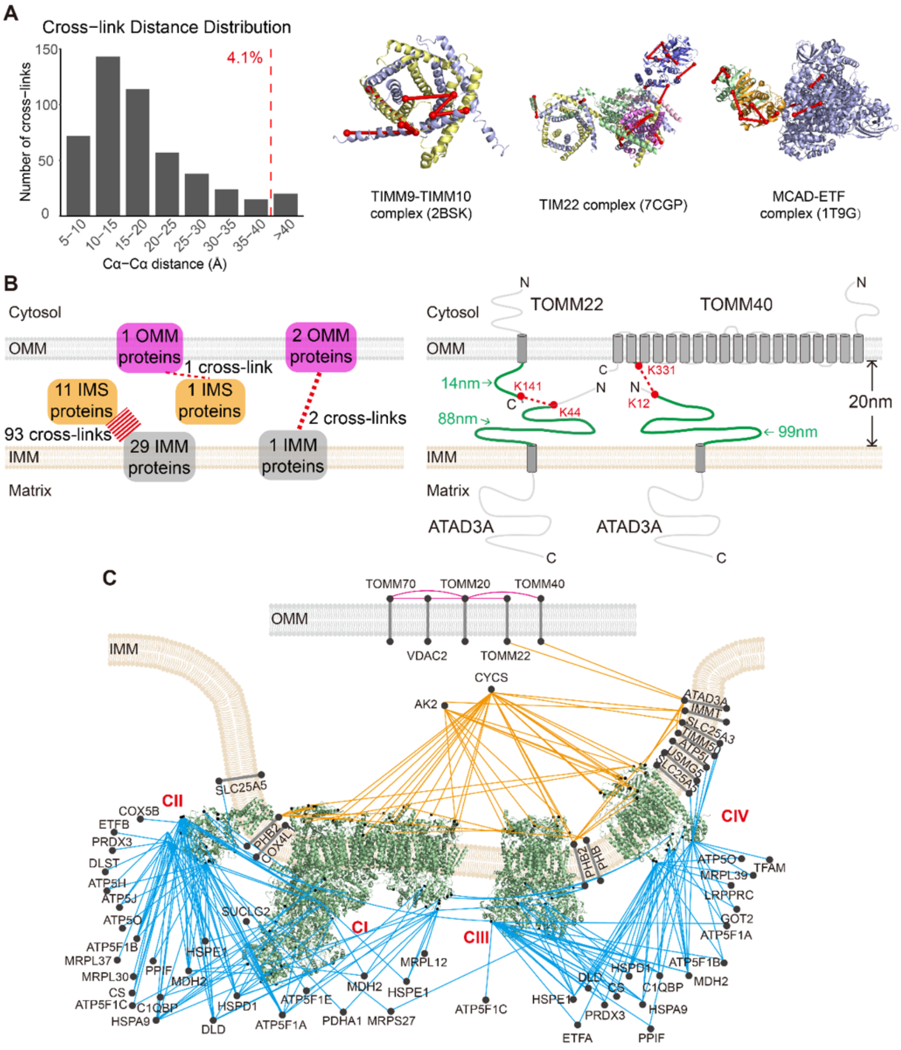

Supplementary Figure 2. DSSO has a maximum labeling radius of 4 nm.

(**A**) The distribution of residue-to-residue distances of cross-links mapped on 31 high-resolution PDB structures. A total of 357 intra-protein links (within the same subunit) and 126 inter-protein links (between different subunits) could be mapped. The selected structures are shown here and in Supplementary Figure 3.

(**B**) Cross-links detected between LMs located in the OMM, IMS and IMM. Only two cross-links between OMM proteins and IMM proteins were detected. These cross-links (TOMM22-K141 – ATAD3A-K44, TOMM40-K331 – ATAD3A-K12) are depicted in the right panel. Both cross-links are located in unstructured protein regions, the maximum length of which is indicated in green. The distance between IMM and OMM is indicated in black[5]. These results suggest that cross-links between OMM and IMM proteins only occur when the linked residues are located in flexible regions that are long enough to reach the other membrane. This finding supports the view that the OMM-IMM distance exceeds the labeling radius of the cross-linker.

(**C**) XL-based sub-network centered at the oxidative phosphorylation complex III and IV. Cross-links are colored according to their sub-compartment localization. Magenta: Cytosolic; Orange: IMS; Blue: matrix.

Source Data are provided as a Source Data file.

Supplementary Figure 3

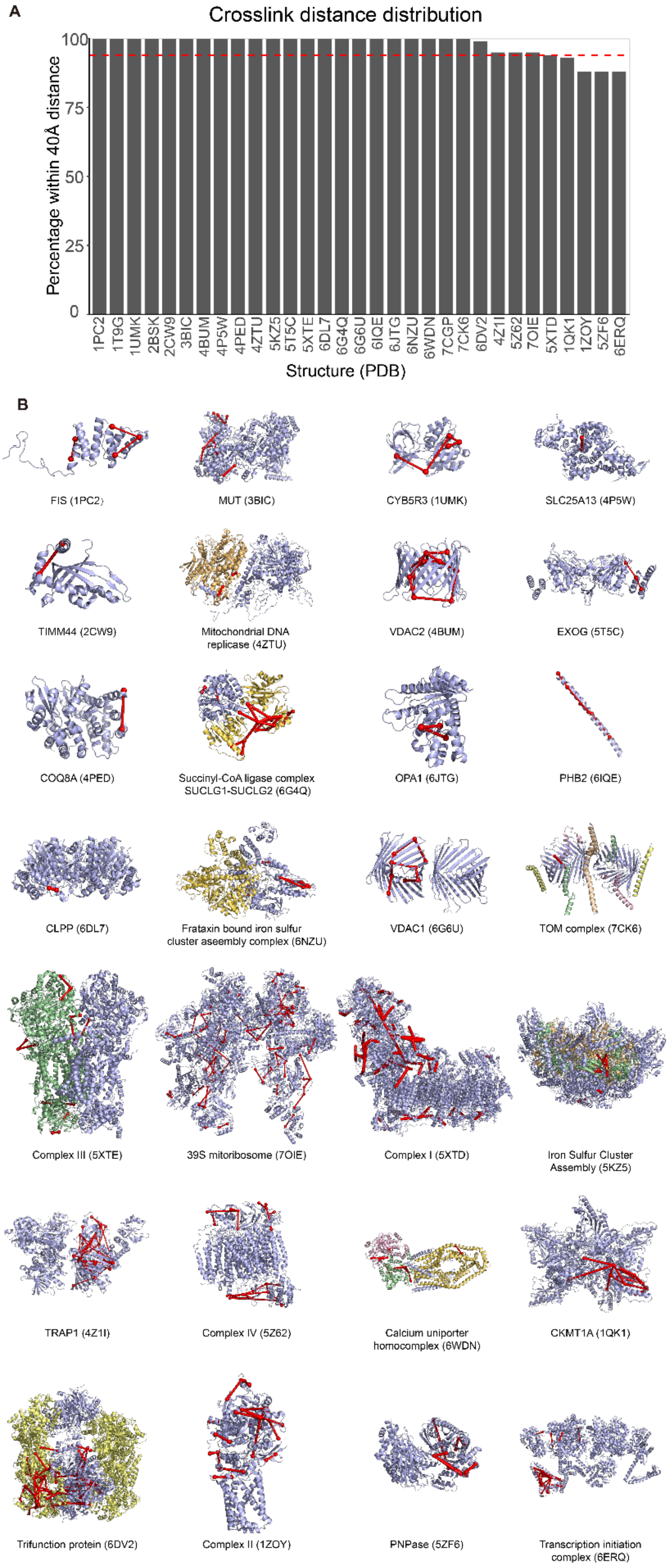

Supplementary Figure 3. Mapping identified cross-links on PDB structures.

(**A**) Percentage of cross-links in each of the 31 analyzed PDB structures that support the 4 nm maximum labeling radius of DSSO-based CLASP.

(**B**) Identified cross-links mapped onto 28 selected high-resolution structures of mitochondrial proteins and complexes. Red lines represent cross-links. The remaining 3 PDB structures are shown in Supplementary Figure 2A. A total of 357 intra-protein links (within the same subunit) and 126 inter-protein links (between different subunits) could be mapped.

Source Data are provided as a Source Data file.

Supplementary Figure 4

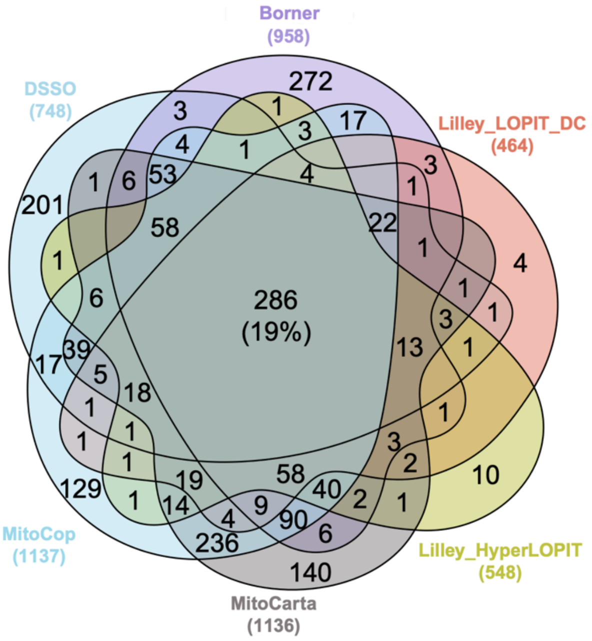

Supplementary Figure 4. Overlap of the mitochondrial proteome generated by DSSO cross-linking with published mitochondrial proteome resources.

The DSSO-cross-linking-based mitochondrial dataset ("DSSO") is compared to the MitoCarta3.0 database ("MitoCarta")[6], the MitoCoP database ("MitoCop")[7], and mitochondrial datasets generated by DOMs ("Borner")[8], LOPIT-DC ("Lilley_LOPIT_DC") and HyperLOPIT (Lilley_HyperLOPIT)[9].

Source Data are provided as a Source Data file.

Supplementary Figure 5

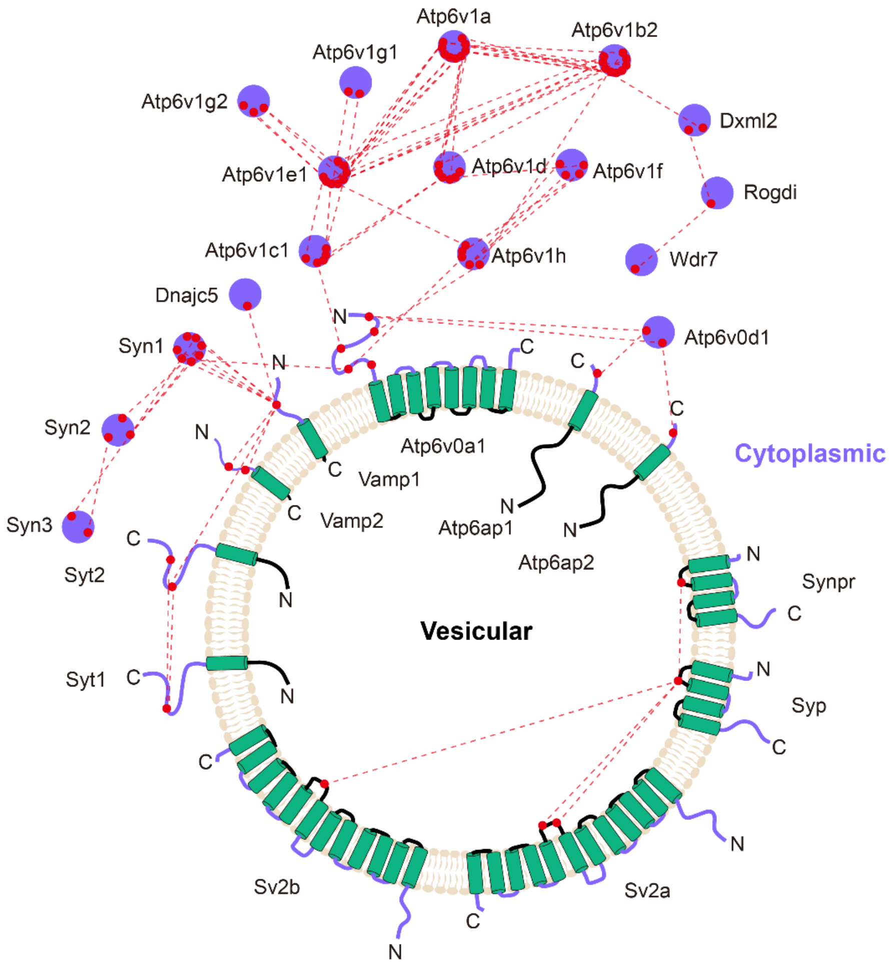

Supplementary Figure 5. Connections among CLASP LMs in synaptic vesicles.

Cross-link based network of SV LMs. Green color indicates transmembrane regions; purple color indicates cytoplasmic regions; black color indicates vesicular regions. Annotations are based on high-resolution structures or literature evidence (summarized in Supplementary Data 7).

Supplementary Figure 6

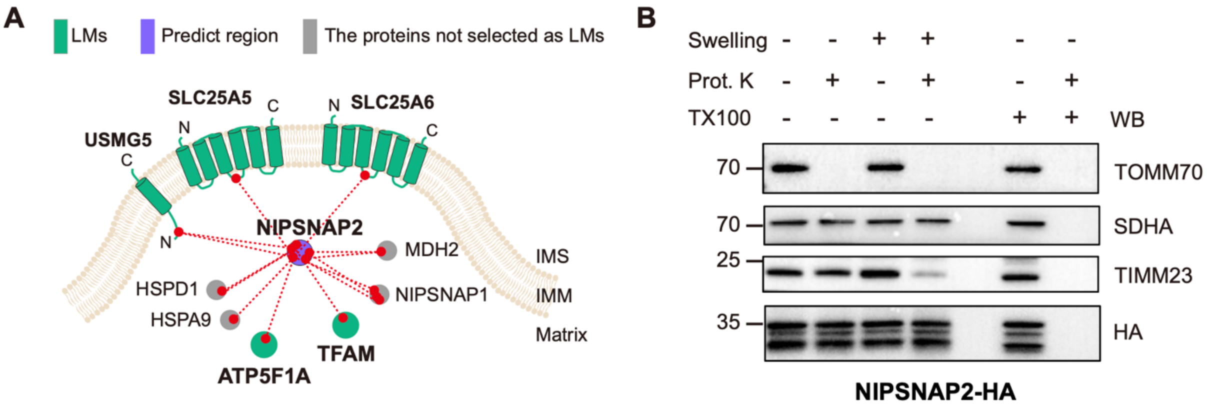

Supplementary Figure 6. Localizing NIPSNAP2 to the mitochondrial matrix.

(A) Cross-link map of NIPSNAP2 and its interacting proteins. LMs are shown in green; NIPSNAP2 is shown in purple. The CLASP annotation of NIPSNAP2 is supported by direct connections to 2 Matrix LMs and 3 IMM LMs. Proteins that are not LMs but support the M localization of NIPSNAP2 are shown in grey. "Predict region" indicates the protein/protein region, for which a CLASP prediction was made.

(B) Protease protection assay to analyze the localization of NIPSNAP2-HA in HEK293T cells. OMM protein TOMM70, IMM protein TIMM23 and matrix protein SDHA are used as markers for each mitochondrial sub-compartment. Locations of molecular weight markers (in kDa) are indicated left of the blots. Experiment was performed once

Source Data are provided as a Source Data file.

Supplementary Figure 7

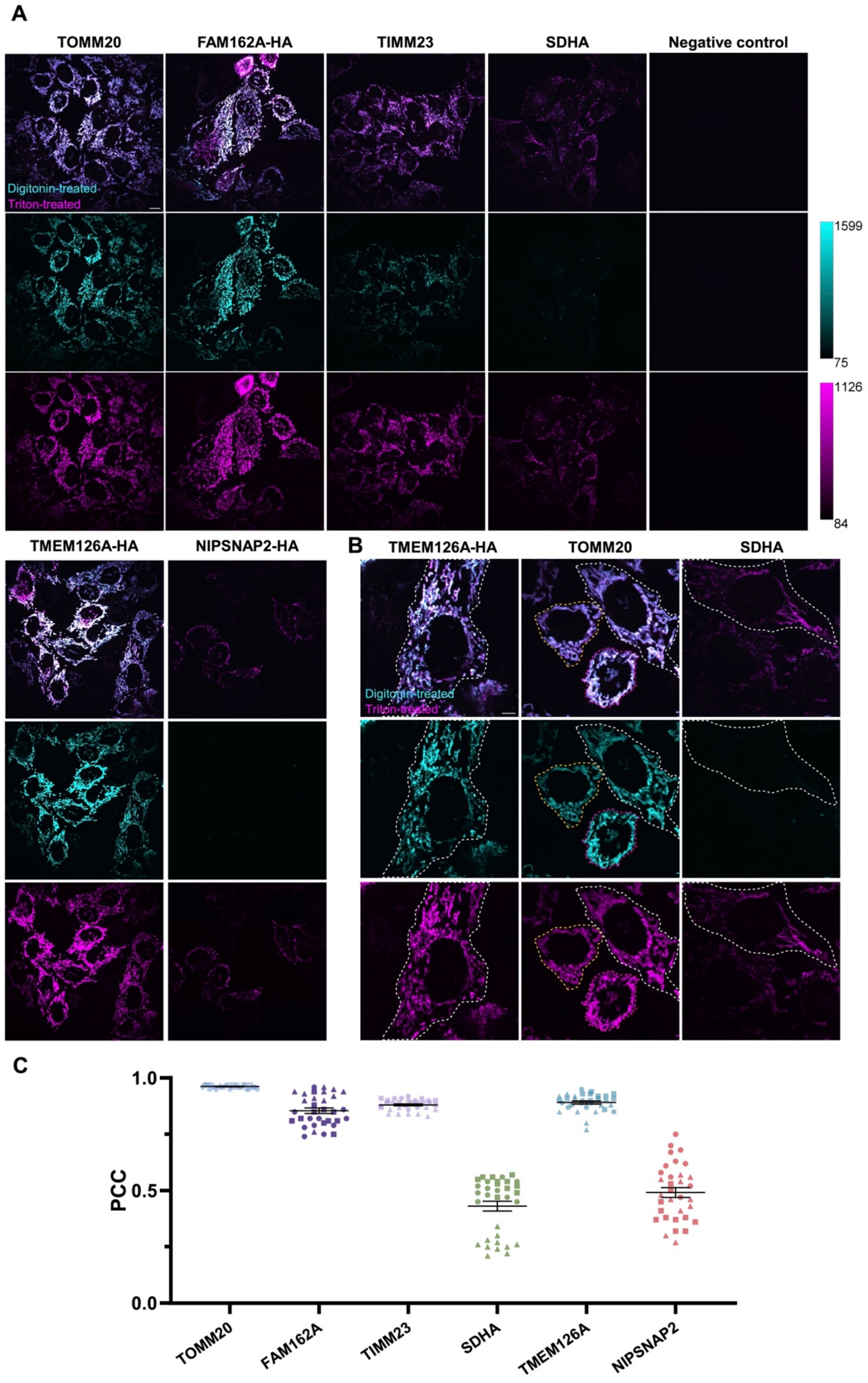

Supplementary Figure 7. Selective permeabilization experiments to determine the sub-compartment localizations of TMEM126A and NIPSNAP2.

(A) Confocal dual-color images after immunofluorescence staining. Each channel shows respective staining of the candidate in dependence of the permeability reagent using highly cross-absorbed secondary

antibodies. Shown are representative images from one out of three biological replicates with antibody and plasmid expressing controls. Image brightness and contrast are adjusted to the same Look-Up Table scale in all images. OMM protein TOMM20, IMM proteins TIMM23 and FAM162A, and matrix protein SDHA are used as markers for each mitochondrial sub-compartment Scale bar: 10 µm.

(B) Representative crops of single cells from the overview images shown in (A). Co-localization analysis was only performed on manually masked out cells within these crops. Masks are shown as dotted white lines. Scale bar: 5 µm. Crop size: 700 x 700 pixels.

(C) Pearson correlation coefficients (PCC) for signal colocalization between digitonin and triton treatment conditions for manually masked out cells within the overview images from (A). Correlation is high for OMM proteins and proteins with antibody binding regions in the IMS since they become accessible under both conditions. Correlation is decreased for proteins with antibody-binding regions in the matrix since they only become available in the presence of triton. NIPSNAP2-HA PCC values resemble those of matrix marker SDHA; TMEM126A PCC values resemble those of IMM markers FAM162A and TIMM23, confirming the CLASP predictions for these proteins. Shown are the mean with the SEM and the individual data points from n=3 independent experiments, distinguishable by the shape of the points (11 images per experiment, with multiple cells each per overview).

Source Data are provided as a Source Data file.

Supplementary Figure 8

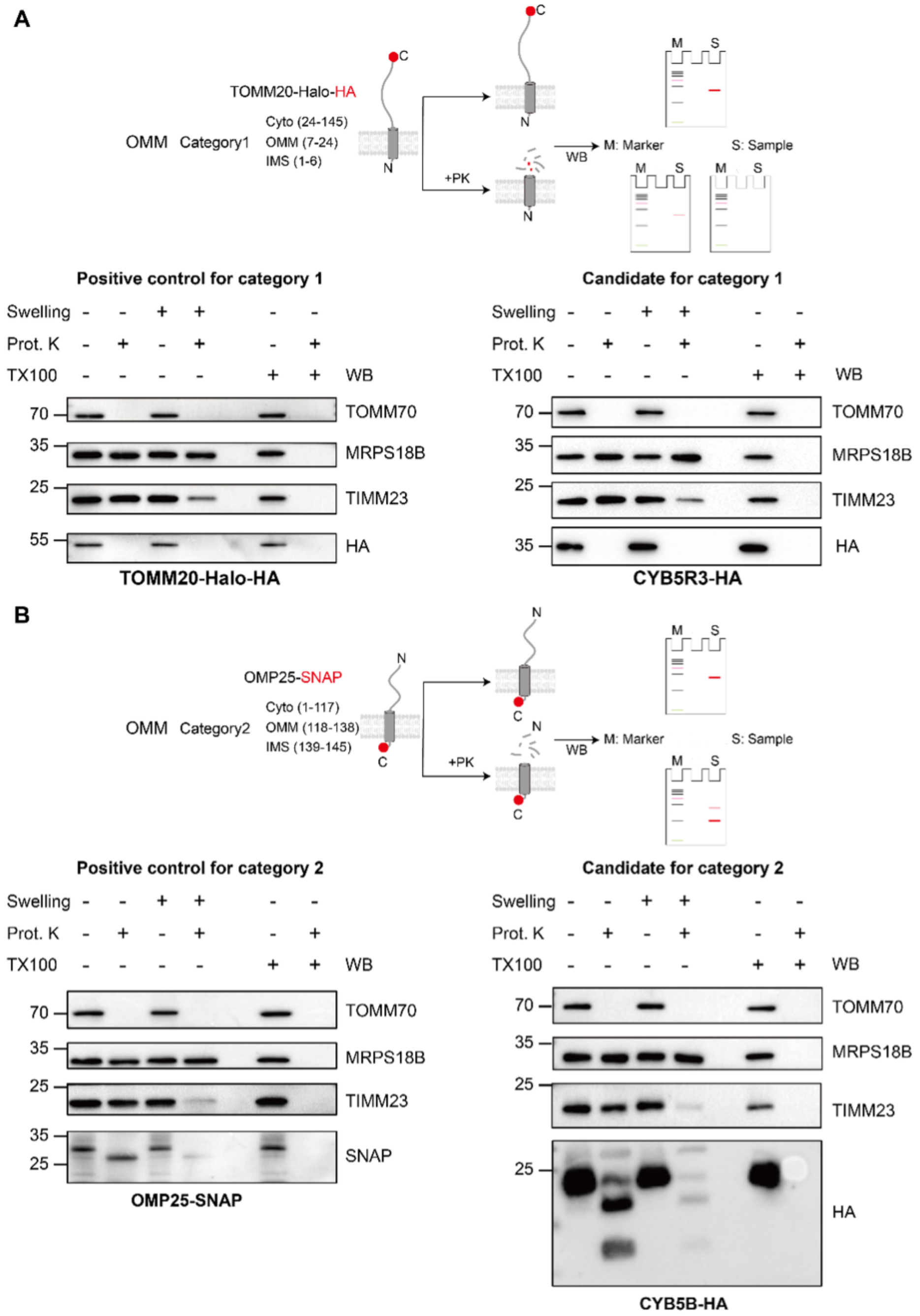

Supplementary Figure 8. CLASP annotates the topologies of CYB5R3 and CYB5B.

(**A**), (**B**) Protease protection assays to analyze the membrane topologies of CYB5R3-HA (A) and CYB5B-HA (B). Depending on the location of the HA-tag, protease (PK) treatment either leads to disappearance of the target protein (category 1 in A) or appearance of a truncated target protein (category 2 in B) on western blot. If proteolysis by PK is incomplete (e.g. for highly abundant target proteins), the undigested protein may still be visible on western blot (partial disappearance of the target protein band in category 1, co-detection of the full-length and truncated target protein in category 2). The OMM proteins TOMM20-Halo-HA and OMP25-SNAP serve as positive controls. Locations of molecular weight markers (in kDa) are indicated left of the blots.

Experiments were performed once. Source Data are provided as a Source Data file.

Supplementary Figure 9

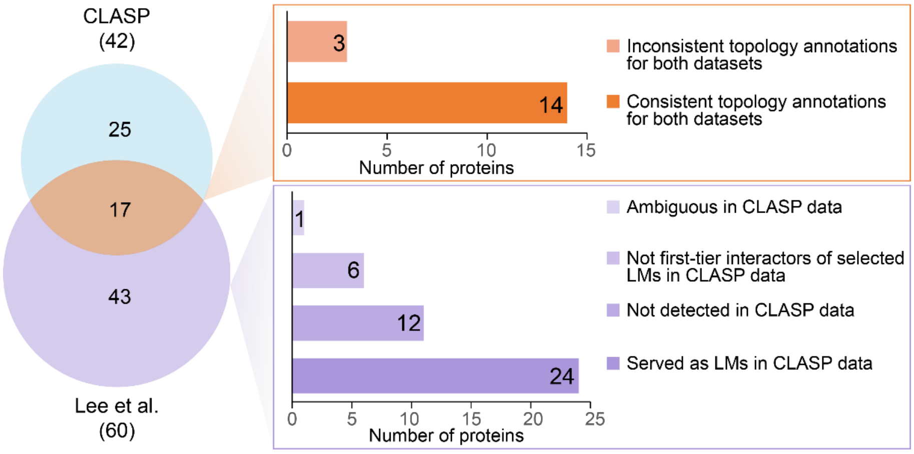

Supplementary Figure 9. Overlap of IMM protein topologies determined by CLASP and in an APEX study by Lee et al. [10]**.** The overlapping and uniquely identified proteins are further annotated in the bar plots on the right.

Supplementary Figure 10

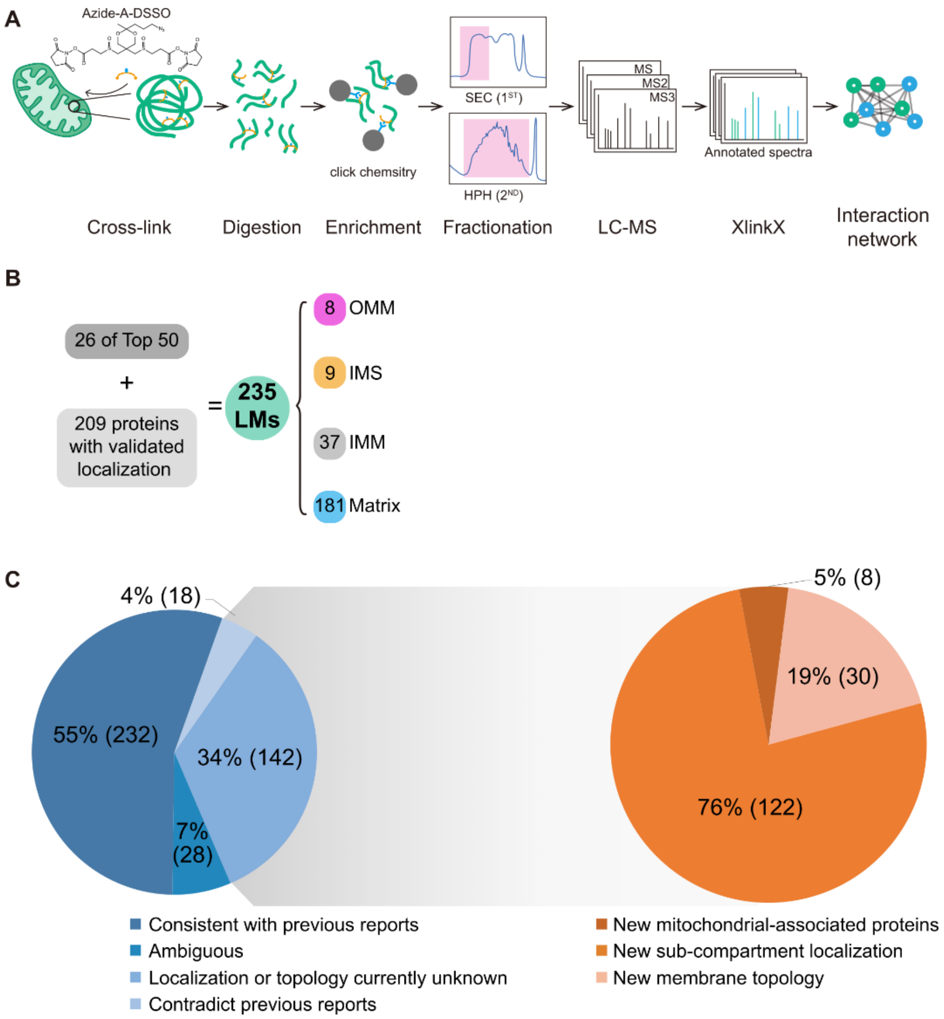

Supplementary Figure 10. Evaluation of CLASP performance in DSBSO dataset.

(**A**) Workflow for the XL-MS analysis of human mitochondrial proteins using DSBSO.

(**B**) The origins and sub-compartment localizations for all 235 LMs for DSBSO dataset.

(**C**) Comparison of CLASP annotations based on the DSBSO dataset to published protein localization information (blue pie chart, left) and breakdown of CLASP annotations that disagree with previous reports or relate to previously unannotated proteins (orange pie chart, right). Annotations of individual proteins are shown in Supplementary Data 10.

Source Data are provided as a Source Data file.

Supplementary Figure 11

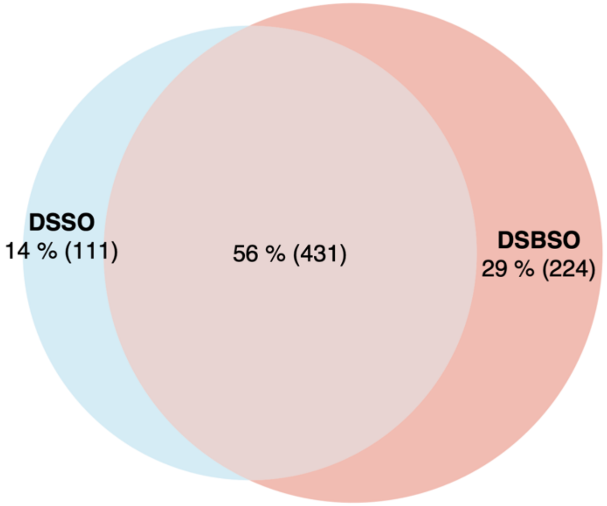

Supplementary Figure 11. Overlap of spatially annotated proteins in DSSO-based CLASP and DSBSO-based CLASP. This plot includes internally consistent LMs (i.e. without conflicting cross-links) and their first-tier interactors.

Supplementary Figure 12

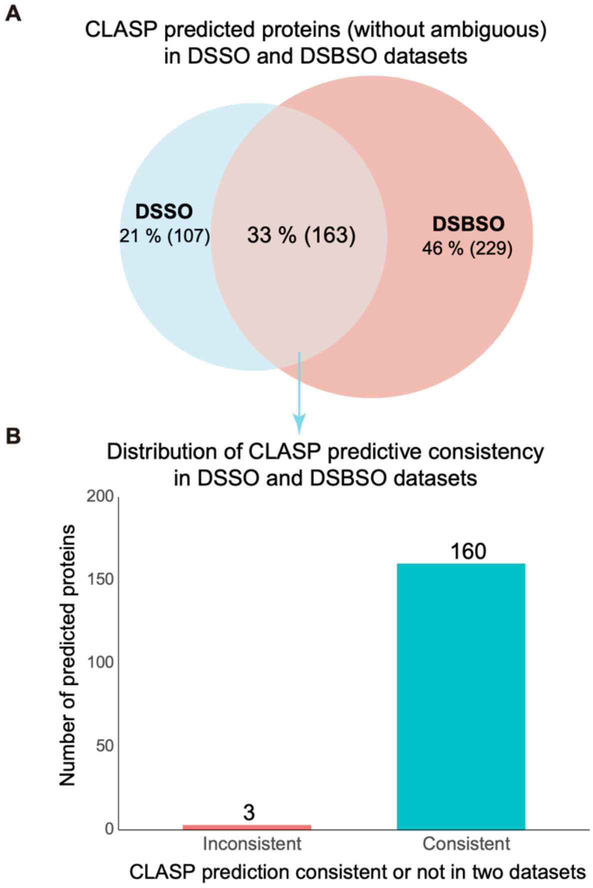

Supplementary Figure 12. Consistency between DSSO-based and DSBSO-based CLASP predictions.

(A) Overlap of proteins, for which unambiguous CLASP predictions could be made using the DSSO and DSBSO mitochondrial XL-MS data.

(B) Number of proteins with consistent and inconsistent prediction results in DSSO-based and DSBSO-based CLASP.

Supplementary Figure 13

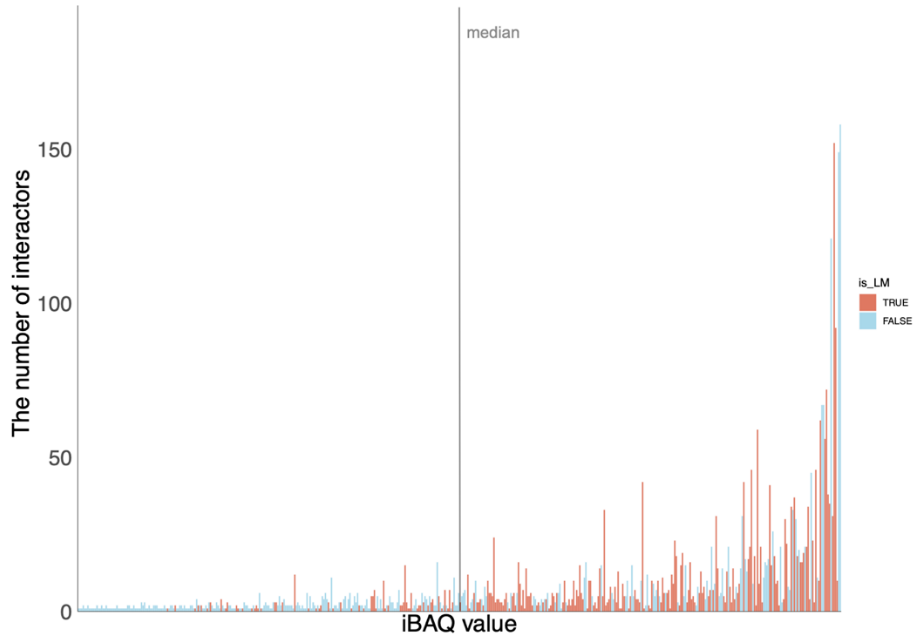

Supplementary Figure 13. Abundance of cross-linked proteins plotted against their number of detected interactors for the DSSO mitochondria dataset.

Abundance is based on the average iBAQ value from three biological replicates. LMs (shown in red) are enriched among higher abundant proteins (i.e., towards the right on the horizontal axis). Higher abundant proteins also tend to have more detected interacting proteins (indicated by longer bars).

Source Data are provided as a Source Data file.

Supplementary Figure 14

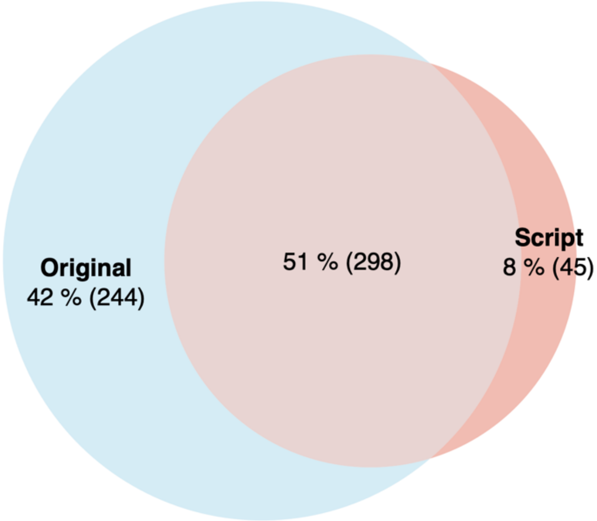

Supplementary Figure 14. Overlap of CLASP LMs and first-tier interactors between the original manual approach and the Python script-based automated CLASP.

The analysis is based on the DSSO mitochondria dataset.

Supplementary Figure 15

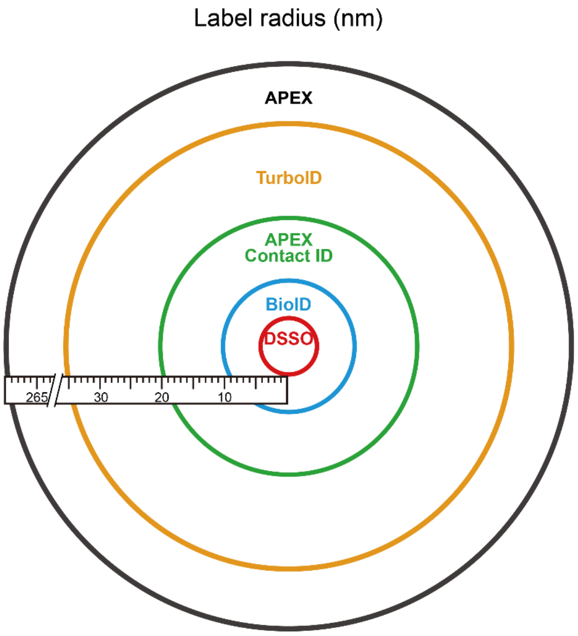

Supplementary Figure 15. Labeling radii of DSSO-based CLASP and other proximity labeling methods.

The following radii are displayed: DSSO: 4 nm (red, shown here), BioID: 10 nm[11] (blue), APEX: 20 nm[12] (green) and 269 ± 41 nm[13] (black), Contact ID: 10-20 nm[14] (green), TurboID: 35 nm[15] (orange).

Supplementary Figure 16

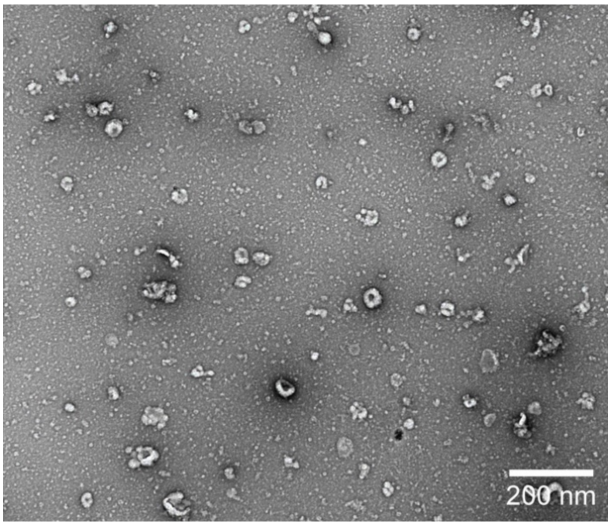

Supplementary Figure 16. Negative stain EM micrograph of SVs prepared following the protocol in this study.

The protocol is described in the Methods section.

Supplementary References

1. Gotze, M., Iacobucci, C., Ihling, C.H. & Sinz, A. A Simple Cross-Linking/Mass Spectrometry Workflow for Studying System-wide Protein Interactions. *Anal Chem* **91**, 10236-10244 (2019).
2. Ihling, C.H., Piersimoni, L., Kipping, M. & Sinz, A. Cross-Linking/Mass Spectrometry Combined with Ion Mobility on a timsTOF Pro Instrument for Structural Proteomics. *Anal Chem* **93**, 11442-11450 (2021).
3. Singh, J. et al. Cross-linking of the endolysosomal system reveals potential flotillin structures and cargo. *Nat Commun* **13**, 6212 (2022).
4. Yilmaz, S., Busch, F., Nagaraj, N. & Cox, J. Accurate and Automated High-Coverage Identification of Chemically Cross-Linked Peptides with MaxLynx. *Anal Chem* **94**, 1608-1617 (2022).
5. Kuhlbrandt, W. Structure and function of mitochondrial membrane protein complexes. *BMC Biol* **13**, 89 (2015).
6. Rath, S. et al. MitoCarta3.0: an updated mitochondrial proteome now with sub-organelle localization and pathway annotations. *Nucleic Acids Res* **49**, D1541-D1547 (2021).
7. Morgenstern, M. et al. Quantitative high-confidence human mitochondrial proteome and its dynamics in cellular context. *Cell Metab* **33**, 2464-2483 e2418 (2021).
8. Schessner, J.P., Albrecht, V., Davies, A.K., Sinitcyn, P. & Borner, G.H.H. Deep and fast label-free Dynamic Organellar Mapping. *Nat Commun* **14**, 5252 (2023).
9. Geladaki, A. et al. Combining LOPIT with differential ultracentrifugation for high-resolution spatial proteomics. *Nat Commun* **10**, 331 (2019).
10. Lee, S.Y. et al. Architecture Mapping of the Inner Mitochondrial Membrane Proteome by Chemical Tools in Live Cells. *J Am Chem Soc* **139**, 3651-3662 (2017).
11. Kim, D.I. et al. Probing nuclear pore complex architecture with proximity-dependent biotinylation. *Proc Natl Acad Sci U S A* **111**, E2453-2461 (2014).
12. Martell, J.D. et al. Engineered ascorbate peroxidase as a genetically encoded reporter for electron microscopy. *Nat Biotechnol* **30**, 1143-1148 (2012).
13. Oakley, J.V. et al. Radius measurement via super-resolution microscopy enables the development of a variable radii proximity labeling platform. *Proc Natl Acad Sci U S A* **119**, e2203027119 (2022).
14. Kwak, C. et al. Contact-ID, a tool for profiling organelle contact sites, reveals regulatory proteins of mitochondrial-associated membrane formation. *Proc Natl Acad Sci U S A* **117**, 12109-12120 (2020).
15. May, D.G., Scott, K.L., Campos, A.R. & Roux, K.J. Comparative Application of BioID and TurboID for Protein-Proximity Biotinylation. *Cells* **9,** 1070 (2020).
