# Supplementary material for: Cross-link assisted spatial proteomics to map sub-organelle proteomes and membrane protein topologies
Source: Nat Commun. 2024 Apr 17;15:3290. doi: 10.1038/s41467-024-47569-x (PMC11024108; doi:10.1038/s41467-024-47569-x)
Supplement: Supplementary file 3 — Description of Additional Supplementary Files [file 41467_2024_47569_MOESM3_ESM.pdf]

Description of Additional Supplementary Files

Supplementary Data 1:
All protein-protein interactions identified in DSSO-cross-linked human mitochondria from three biological replicates, filtered by 2% FDR at unique cross-linked residue pairs level.

Supplementary Data 2:
All protein-protein interactions identified in DSSO-cross-linked human mitochondria from the first biological replicate, filtered by 2% FDR at unique cross-linked residue pairs level.

Supplementary Data 3:
All protein-protein interactions identified in the DSSO-cross-linked mitochondria from the second biological replicate, filtered by 2% FDR at unique cross-linked residue pairs level.

Supplementary Data 4:
All protein-protein interactions identified in DSSO-cross-linked human mitochondria from the third biological replicate, filtered by 2% FDR at unique cross-linked residue pairs level.

Supplementary Data 5:
Localization markers (LMs) and their first-tier interactors for DSSO-cross-linked human mitochondria.

Supplementary Data 6:
All protein-protein interactions identified in synaptic vesicles from mouse brains, filtered by 2% FDR at unique cross-linked residue pairs level.

Supplementary Data 7:
Synaptic vesicles localization markers (LMs).

Supplementary Data 8:
Primer sequence list.

Supplementary Data 9:
All protein-protein interactions identified in DSBSO-cross-linked human mitochondria, filtered by 2% FDR at unique cross-linked residue pairs level.

Supplementary Data 10:
Localization markers (LMs) and their first-tier interactors for mitochondria of DSBSO dataset.

Supplementary Data 11:
Output from the Python tool for automated CLASP prediction when applied to the dataset of DSSO-cross-linked mitochondria.
