# Supplementary material for: Cross-link assisted spatial proteomics to map sub-organelle proteomes and membrane protein topologies
Source: Nat Commun. 2024 Apr 17;15:3290. doi: 10.1038/s41467-024-47569-x (PMC11024108; doi:10.1038/s41467-024-47569-x)
Supplement: Supplementary file 5 — Reporting Summary [file 41467_2024_47569_MOESM5_ESM.pdf]

Corresponding author(s): Fan Liu

Last updated by author(s): Mar 18, 2024

Reporting Summary

Nature Portfolio wishes to improve the reproducibility of the work that we publish. This form provides structure for consistency and transparency in reporting. For further information on Nature Portfolio policies, see our Editorial Policies and the Editorial Policy Checklist.

Statistics

For all statistical analyses, confirm that the following items are present in the figure legend, table legend, main text, or Methods section.

n/a	Confirmed	
☐	☒	The exact sample size (n) for each experimental group/condition, given as a discrete number and unit of measurement
☐	☒	A statement on whether measurements were taken from distinct samples or whether the same sample was measured repeatedly
☒	☐	The statistical test(s) used AND whether they are one- or two-sided<br>*Only common tests should be described solely by name; describe more complex techniques in the Methods section.*
☒	☐	A description of all covariates tested
☒	☐	A description of any assumptions or corrections, such as tests of normality and adjustment for multiple comparisons
☐	☒	A full description of the statistical parameters including central tendency (e.g. means) or other basic estimates (e.g. regression coefficient) AND variation (e.g. standard deviation) or associated estimates of uncertainty (e.g. confidence intervals)
☒	☐	For null hypothesis testing, the test statistic (e.g. F, t, r) with confidence intervals, effect sizes, degrees of freedom and P value noted<br>*Give P values as exact values whenever suitable.*
☒	☐	For Bayesian analysis, information on the choice of priors and Markov chain Monte Carlo settings
☒	☐	For hierarchical and complex designs, identification of the appropriate level for tests and full reporting of outcomes
☐	☒	Estimates of effect sizes (e.g. Cohen's d, Pearson's r), indicating how they were calculated

Our web collection on statistics for biologists contains articles on many of the points above.

Software and code

Policy information about availability of computer code

Data collection	Orbitrap Fusion Tune Application (v3.4), Orbitrap Fusion Lumos Tune Application (v3.4), Xcalibur (v4.4) - all Thermo Scientific / NIS-Elements (Nikon) / Micro-Manager (https://micro-manager.org/)
Data analysis	XlinkX stand-alone (Liu et al., Nat Commun, 2017) / RStudio (2021.09.0) / R (v4.1.1) / TMHMM (v2.0, DTU Health Tech) /Pymol (v2.1.0, Schrodinger LLC) / Proteome Discoverer (v2.4, Thermo Scientific) / MaxQuant (v1.6.2a, MPI of Biochemistry) / Cytoscape (v3.8.2, https://cytoscape.org/) / FIJI (https://imagej.net/software/fiji/) / Coloc2 (https://imagej.net/plugins/coloc-2) / Clone Manager (v8.0, Sci-Ed) / Image Lab (v6.0.1, Bio-Rad)<br>The Python tool for automated CLASP, a user guide, and example input and output data are available through GiHub (https://github.com/theliulab/protein_location_prediction) and Zenodo (https://doi.org/10.5281/zenodo.10824759). The Github deposition is freely available under a MIT license; the Zenodo deposition is freely available under a Creative Commons Attribution 4.0 International license.

For manuscripts utilizing custom algorithms or software that are central to the research but not yet described in published literature, software must be made available to editors and reviewers. We strongly encourage code deposition in a community repository (e.g. GitHub). See the Nature Portfolio guidelines for submitting code & software for further information.

Data

Policy information about availability of data

All manuscripts must include a data availability statement. This statement should provide the following information, where applicable:

- Accession codes, unique identifiers, or web links for publicly available datasets
- A description of any restrictions on data availability
- For clinical datasets or third party data, please ensure that the statement adheres to our policy

All CLASP predictions and LMs are reported in Supplementary Data 5 (DSSO-cross-linked mitochondria, manual CLASP), 7 (SVs), 10 (DSBSO-cross-linked mitochondria), and 11 (DSSO-cross-linked mitochondria, automated CLASP). The underlying cross-link identifications are reported in Supplementary Data 1-4 (DSSO-cross-linked mitochondria), 6 (SVs), and 9 (DSBSO-cross-linked mitochondria). The mass spectrometry raw data have been deposited to the PRIDE repository with the dataset identifiers PXD032132 [https://www.ebi.ac.uk/pride/archive/projects/PXD032132] and PXD046382 [https://www.ebi.ac.uk/pride/archive/projects/PXD046382].
For validating the mitochondrial localization of endogenous Fam136A in human cells, we used publicly available data from Human Protein Atlas (https://www.proteinatlas.org/ENSG00000035141-FAM136A/subcellular#human).
For comparing CLASP to published spatial proteomics studies, we used
- The Go et al. dataset (Supp. Table 9 of the original paper 7)
- The Antonicka et al. dataset (Table S4 in 29)
- The Lee et al. dataset (Data Set S7 of 26)
- The DOMs dataset from Schessner et al. (Supp. Data 1 in 35)
- The LOPIT-DC and HyperLOPIT datasets from Geladaki et al. (Supp. Data 1 and 2 in 36)
- The MitoCoP database from Morgenstern et al. (Table S1 in 34)
- The MitoCarta3.0 database described by Rath et al.33 (available at https://www.broadinstitute.org/files/shared/metabolism/mitocarta/human.mitocarta3.0.html)
For confirming the labeling radius of DSSO-based CLASP, the following publicly available PDB structures were used:
- the mitochondrial electron transport chain complexes I, II, III and IV with PDB accession codes 5XTD [https://doi.org/10.2210/pdb5XTD/pdb], 1ZOY [https://doi.org/10.2210/pdb1ZOY/pdb], 5XTE [https://doi.org/10.2210/pdb5XTE/pdb] and 5Z62 [https://doi.org/10.2210/pdb5Z62/pdb], respectively
- TOMM complex with PDB accession code 7CK6 [https://doi.org/10.2210/pdb7CK6/pdb]
- TIMM22 complex with PDB accession code 7CGP [https://doi.org/10.2210/pdb7CGP/pdb]
- TIMM9-TIMM10 complex with PDB accession code 2BSK [https://doi.org/10.2210/pdb2BSK/pdb]
- succinyl-CoA ligase complex SUCLG1-SUCLG2 with PDB accession code 6G4Q [https://doi.org/10.2210/pdb6G4Q/pdb]
- MCAD-ETF complex with PDB accession code 1T9G [https://doi.org/10.2210/pdb1T9G/pdb]
- 39S mitoribosome with PDB accession code 7OIE [https://doi.org/10.2210/pdb7OIE/pdb]
- frataxin bound iron sulfur cluster assembly complex with PDB accession code 6NZU [https://doi.org/10.2210/pdb6NZU/pdb]
- iron sulfur cluster assembly with PDB accession code 5KZ5 [https://doi.org/10.2210/pdb5KZ5/pdb]
- calcium uniporter homocomplex with PDB accession code 6WDN [https://doi.org/10.2210/pdb6WDN/pdb]
- transcription ignition complex with PDB accession code 6ERQ [https://doi.org/10.2210/pdb6ERQ/pdb]
- mitochondrial DNA replicase with PDB accession code 4ZTU [https://doi.org/10.2210/pdb4ZTU/pdb]
- trifunction protein with PDB accession code 6DV2 [https://doi.org/10.2210/pdb6DV2/pdb]
- FIS with PDB accession code 1PC2 [https://doi.org/10.2210/pdb1PC2/pdb]
- CYB5R3 with PDB accession code 1UMK [https://doi.org/10.2210/pdb1UMK/pdb]
- TIMM44 with PDB accession code 2CW9 [https://doi.org/10.2210/pdb2CW9/pdb]
- MUT with PDB accession code 3BIC [https://doi.org/10.2210/pdb3BIC/pdb]
- VDAC1 with PDB accession code 6G6U [https://doi.org/10.2210/pdb6G6U/pdb]
- VDAC2 with PDB accession code 4BUM [https://doi.org/10.2210/pdb4BUM/pdb]
- SLC25A13 with PDB accession code 4P5W [https://doi.org/10.2210/pdb4P5W/pdb]
- COQ8A with PDB accession code 4PED [https://doi.org/10.2210/pdb4PED/pdb]
- EXOG with PDB accession code 5T5C [https://doi.org/10.2210/pdb5T5C/pdb]
- CLPP with PDB accession code 6DL7 [https://doi.org/10.2210/pdb6DL7/pdb]
- PHB2 with PDB accession code 6IQE [https://doi.org/10.2210/pdb6IQE/pdb]
- OPA1 with PDB accession code 6JTG [https://doi.org/10.2210/pdb6JTG/pdb]
- TRAP1 with PDB accession code 4Z1L [https://doi.org/10.2210/pdb4Z1L/pdb]
- CKMT1A with PDB accession code 1QK1 [https://doi.org/10.2210/pdb1QK1/pdb]
- PNPase with PDB accession code 5FZ6 [https://doi.org/10.2210/pdb5FZ6/pdb]
A source data file is provided with this manuscript.

Human research participants

Policy information about studies involving human research participants and Sex and Gender in Research.

Reporting on sex and gender	Research did not involve human participants.
Population characteristics	Research did not involve human participants.
Recruitment	Research did not involve human participants.
Ethics oversight	Research did not involve human participants.

Note that full information on the approval of the study protocol must also be provided in the manuscript.

Field-specific reporting

Please select the one below that is the best fit for your research. If you are not sure, read the appropriate sections before making your selection.

☒ Life sciences ☐ Behavioural & social sciences ☐ Ecological, evolutionary & environmental sciences

For a reference copy of the document with all sections, see nature.com/documents/nr-reporting-summary-flat.pdf

Life sciences study design

All studies must disclose on these points even when the disclosure is negative.

Sample size	Samples sizes were chosen based on preliminary experiments and common practice in the field. No statistical method for sample size calculation was used. Sample sizes are sufficient to assess technical and biological variability of the results.
Data exclusions	No data were excluded from our analyses.
Replication	All attempts at replication were successful.<br>The experiments to generate the main dataset of this study (DSSO cross-linking of mitochondria) was performed in 3 biological replicates. The replicates showed the expected variability and the results and concepts were additionally confirmed by DSBSO cross-linking experiments of mitochondria and synaptic vesicles (both n=1 because the main purpose of these experiments was to orthogonally validate different aspects the replicated main dataset).<br>Pearson correlation analyses of the imaging data were performed on 4 biological replicates (FAF2 localizatio analysis) or 3 biological replicates (Selective permeabilization experiments).<br>All other experiments were done once - in lieu of replication, these results were confirmed by complementary imaging-based, immunoblot-based and/or mass spectrometry-based assays.
Randomization	This is a molecular and structural biology study. There were no experimental groups for which randomization would be relevant.
Blinding	This is a molecular and structural biology study. There was no group allocation for which blinding would be relevant.

Reporting for specific materials, systems and methods

We require information from authors about some types of materials, experimental systems and methods used in many studies. Here, indicate whether each material, system or method listed is relevant to your study. If you are not sure if a list item applies to your research, read the appropriate section before selecting a response.

Materials & experimental systems

n/a	Involved in the study	
☐	☒	Antibodies
☐	☒	Eukaryotic cell lines
☒	☐	Palaeontology and archaeology
☐	☒	Animals and other organisms
☒	☐	Clinical data
☒	☐	Dual use research of concern

Methods

n/a	Involved in the study	
☒	☐	ChIP-seq
☒	☐	Flow cytometry
☒	☐	MRI-based neuroimaging

Antibodies

Antibodies used	The following antibodies were used for western blotting: anti-HA (1:1000, mouse, Abcam, ab18181); anti-SNAP (1:1000, rabbit, NEB, P9310S); anti-MRPS18B, anti-TIMM44 and TOMM70 (1:500, 1:1000, 1:500, rabbit, ProteinTech, 16139-1-AP, 13859-1-AP, 14528-1-AP); anti-TIMM23 (1:1000, mouse, DB Biotech, 611222). After washing 3 times with TBST, secondary antibody peroxidase-conjugated affinipure goat anti- mouse lgG and peroxidase-conjugated affinipure goat anti-rabbit lgG (H+L, Jackson ImmunoResearch, 115-035-003 and 111-035-144).<br>The following primary antibodies were used in FAF2 experiment: anti-mouse-TOMM20 (1:100, Santa Cruz, sc-17764, RRID:AB 628381), anti-rat-HA (1:100, Chromotek, 7c9-100, RRID:AB_2631399), anti-rabbit-Calreticulin (1:100, Thermo, PA3-900, RRID:AB_325990), anti-rabbit-FAF2 (Proteintech, 16251-1-AP, RRID:AB_2262469)<br>The following primary antibodies were used in FAM136A experiment: anti-mouse-TOMM20 (1:100, Santa Cruz, sc-17764, RRID:AB_628381), anti-rabbit-HA (1:100, Cayman, cay162200-1, RRID:AB_327903).<br>The following primary antibodies were used in selective permeabilization experiment: anti-mouse-TOMM20 (1:100, Santa Cruz, sc-17764, RRID:AB_628381), anti-mouse-HA (1:100, Santa Cruz, sc-7392, RRID:AB_627809), anti-mouse-TIMM23 (1:50, BD Biosciences, 611222, RRID:AB_398754), anti-rabbit-COX4 (1:100, Cell Signaling, 4850, RRID:AB 2085424), anti-mouse-SDHA (1:50, Abcam, ab14715, RRID:AB 301433)<br>The following secondary antibodies were used in FAF2 and FAM136A experiment: anti-rat-AF488 (1:200, Invitrogen, A11006, RRID:AB_2534074), anti-mouse-AF594 (1:200, Thermo, A11032, RRID:AB_2534091) and anti-rabbit-AF647 (1:200, Thermo, A21244,

RRID:AB_2535812).
The following secondary antibodies were used in selective permeabilization experiment: anti-mouse- CF®568 (Biotum, 20105, RRID:AB 10557030), anti-rabbit-CF®568 (Biotum, 20098, RRID:AB 10557118), anti-mouse-CF®640R (Biotum, 20177, RRID:AB 10853623), anti-rabbit-CF®640R (Biotum, 20178, RRID:AB 10852688).

Validation

anti-HA, PMID: 33377142, https://www.abcam.com/products/primary-antibodies/ha-tag-antibody-hac5-ab18181.html; anti-SNAP, PMID: 36173346, https://international.neb.com/products/p9310-anti-snap-tag-antibody-polyclonal#Product%20Information; anti-MRPS18B, PMID: 27693358, https://www.ptglab.com/products/MRPS18B-Antibody-16139-1-AP.htm; anti-TIMM44, PMID: 27693358, https://www.ptglab.com/products/TIMM44-Antibody-13859-1-AP.htm; anti-TOMM70, PMID: 31618756, https://www.ptglab.com/products/TOM70-Antibody-14528-1-AP.htm; anti-TIMM23, PMID: 10393182, https://www.bdbiosciences.com/en-de/products/reagents/microscopy-imaging-reagents/immunofluorescence-reagents/purified-mouse-anti-tim23.611222; peroxidase-conjugated affinipure goat anti- mouse lgG, PMID: 36895943, https://www.jacksonimmuno.com/catalog/products/115-035-003; peroxidase-conjugated affinipure goat anti-rabbit lgG, PMID: 36830070, https://www.jacksonimmuno.com/catalog/products/111-035-144; anti-mouse-TOMM20, PMID: 36270994, https://www.scbt.com/p/tom20-antibody-f-10; anti-rat-HA, PMID: 32359443, https://www.ptglab.com/products/HA-antibody-7C9.htm; anti-rabbit-Calreticulin,PMID: 29691234, https://www.thermofisher.com/antibody/product/Calreticulin-Antibody-Polyclonal/PA3-900;anti-rabbit-HA, validated for human cells for applications of WB, https://www.caymanchem.com/product/162200/ha-polyclonal-antibody; anti-rat-AF594, PMID: 35787744, https://www.thermofisher.com/antibody/product/Goat-anti-Rat-IgG-H-L-Cross-Adsorbed-Secondary-Antibody-Polyclonal/A-11007; anti-mouse-AF594, PMID: 31430047, https://www.thermofisher.com/antibody/product/Goat-anti-Mouse-IgG-H-L-Highly-Cross-Adsorbed-Secondary-Antibody-Polyclonal/A-11032; anti rabbit-AF647, PMID: 31388010, https://www.thermofisher.com/antibody/product/Goat-anti-Rabbit-IgG-H-L-Cross-Adsorbed-Secondary-Antibody-Polyclonal/A-21244;
anti-mouse-AF594, PMID: 31430047, https://www.thermofisher.com/antibody/product/Goat-anti-Mouse-IgG-H-L-Highly-Cross-Adsorbed-Secondary-Antibody-Polyclonal/A-11032.
Validation data for all other antibodies are available on the manufacturers' websites.

Eukaryotic cell lines

Policy information about cell lines and Sex and Gender in Research

Cell line source(s)

HEK293T (ATCC,CRL-3216)
HeLa (ATCC, CCL-2)
HeLa-COX8A-SNAP (Cell line was kindly donated by authors from Stephan, T., Roesch, A., Riedel, D. & Jakobs, S. Live-cell STED nanoscopy of mitochondrial cristae. Sci Rep 9 (2019))

Authentication

Cell lines were not further authenticated.

Mycoplasma contamination

All cell lines were tested negative for mycoplasma contamination.

Commonly misidentified lines
(See ICLAC register)

No commonly misidentified lines were used in this study.

Animals and other research organisms

Policy information about studies involving animals; ARRIVE guidelines recommended for reporting animal research, and Sex and Gender in Research

Laboratory animals

Synaptic vesicles were obtained from mice brain (C57BL/6, male and female mice at 6 weeks of age). Mice were kept in groups of up to six animals in standard individually ventilated cages of 524 cm2 at (21 +/- 2)°C, (50 +/- 10)% relative humidity, and a 12:12 h light–dark cycle. Cages contained bedding and nesting material. Food and water were provided ad libitum.

Wild animals

No wild animals were used in this study.

Reporting on sex

Sex was not considered in this study because only brains were used for the synaptic vesicle purification.

Field-collected samples

No field-collected samples were used in this study.

Ethics oversight

All animal experiments were reviewed and approved by the ethics committee of the “Landesamt für Gesundheit und Soziales” (LAGeSo) Berlin and were conducted according to the committee’s guidelines under animal experimentation permits. At the institute, the Animal Care Officer and the LAGeSo monitored compliance with all regulations.

Note that full information on the approval of the study protocol must also be provided in the manuscript.
